# Radiographic, Ultrasonographic and Shear Elastosonographic Changes in Patellar Ligament in Dogs Undergoing Tibial Plateau Leveling Osteotomy

**DOI:** 10.3390/vetsci12080745

**Published:** 2025-08-11

**Authors:** Angela Palumbo Piccionello, Luca Pennasilico, Adolfo Maria Tambella, Sara Sassaroli, Margherita Galosi, Nicola Pilati, Fabrizio Dini

**Affiliations:** 1 School of Bioscience and Veterinary Medicine, University of Camerino, 62024 Matelica, Italy; angela.palumbo@unicam.it (A.P.P.); adolfomaria.tambella@unicam.it (A.M.T.); margherita.galosi@unicam.it (M.G.); nicola.pilati@unicam.it (N.P.); fabrizio.dini@unicam.it (F.D.); 2 Futuravet, Veterinary Referral Center, 62929 Tolentino, Italy

**Keywords:** patellar ligament, elastosonography, tibial plateau leveling osteotomy, cranial cruciate ligament rupture, dog

## Abstract

This study aims to evaluate the morpho-functional changes in the patellar ligament in dogs undergoing tibial plateau leveling osteotomy (TPLO) during the first six months of the postoperative follow-up and correlate the radiographic and conventional ultrasonography changes in the patellar ligament with its elastic properties. Dogs with a unilateral cranial cruciate ligament (CCL) rupture who underwent TPLO were enrolled. The patients were subjected to radiographic, ultrasonographic and elastosonographic evaluations of the patellar ligament before surgery (T0) and 1 month (T1), 2 months (T2) and 6 months (T3) after surgery. The radiographic and ultrasound thicknesses of the patellar ligament were recorded at three points: proximal, central and distal. A color map of strain elastosonography with superimposed B-mode images was used to evaluate the elasticity (hardness and softness) of the ligament. Twenty-two dogs completed the trial. At T0, the patellar ligament showed statistically reduced radiographic and ultrasonographic thicknesses compared to those at T1, T2 and T3. Additionally, the patellar ligament in the distal portion was statistically thicker in comparison to the proximal and central points at T2 and T3. At T1, T2 and T3, there was an increase in hardness and a reduction in softness of the patellar ligament compared to that at T0. The elastosonographic analysis and the radiographic and ultrasonographic thicknesses of the patellar ligament showed no correlation. The patellar ligament showed structural and mechanical alterations in the dogs undergoing TPLO, even six months after surgery.

## Simple Summary

The thickening and the desmopathy of the patellar ligament are two of the reported complications following tibial plateau leveling osteotomy (TPLO). This study aims to evaluate the appearance of the patellar ligament using radiographic, ultrasound and elastosonography on dogs undergoing TPLO during short- and long term follow-ups and correlate the radiographic and ultrasonography alterations in the patellar ligament with its elasticity. The results highlight the presence of structural and mechanical changes in the patellar ligament in subjects undergoing TPLO before and over six months after surgery. The radiographic and ultrasound examinations provide similar results in the evaluation of the thickness of the patellar ligament, while elastosonography did not show a correlation with the other two techniques.

## 1. Introduction

A cranial cruciate ligament (CCL) rupture is a common orthopedic injury in dogs [1]. The insufficiency of this ligament causes multiplanar stifle instability and subsequent secondary injuries of other anatomical structures, including the medial meniscus, the medial collateral ligament and the patellar ligament [2,3,4,5].

To restore the stability of the joint, this pathology requires a surgical treatment [6]. Tibial plateau leveling osteotomy (TPLO) and tibial tuberosity advancement (TTA) represent the most common surgical procedures to stabilize the joint [7], and they are associated with satisfactory outcomes [8,9].

Although TPLO is a standardized surgery, it is not without complications [10,11,12,13,14]. One of the reported complications is the thickening and the desmopathy of the patellar ligament following tibial osteotomies like TPLO and TTA [15,16,17]. The etiopathogenetic hypothesis is that these two techniques modify the biomechanics of the stifle. Some authors have found that after TPLO, the patella moves slightly cranially with respect to the trochlear groove, and in some cases also distally [18,19,20]. In fact, it has been described that TPLO reduces the moment arm of the stifle extensor mechanism; therefore, the patellar ligament requires a greater force to achieve the same torque, which results in continuous stress on it [15].

In these studies, it was highlighted that the canine patellar ligament showed radiographically detectable thickening in follow-ups after TPLO or TTA [15,17]. In addition, the ultrasound aspect of the patellar ligament showed fiber disruption, changes in echogenicity and periligamentous hyperechogenicity [16]. However, these alterations were often not correlated to clinical signs [15,16,17].

Recently, other authors described the detection of patellar ligament changes even in dogs affected by CCL rupture who had not yet received treatment. These alterations worsened over the time between the onset of CCL rupture and the day of diagnosis, suggesting that the uncontrolled cranial tibial translation could have stressed this ligament [21].

In recent years, a relatively novel imaging technique known as elastosonography has gained popularity for detecting ligament and tendinous diseases in both human and veterinary medicine [22,23,24,25]. Unlike the conventional ultrasonographic techniques, elastosonography allows for the assessment of the mechanical properties of ligaments and tendons, providing information on the elasticity (softness and hardness) of these structures. Based on this rationale, elastosonography provides qualitative and semiquantitative evaluations related to the function of tendons and ligaments [26,27].

Palumbo Piccionello et al. (2018) showed that the patellar ligaments of healthy dogs were predominantly soft [28]. To perform assessments, the authors used real-time strain elastosonography, which measures tissue deformation in response to an external force and its capability to return to its original shape. Similarly, the authors highlighted an increase in hardness and a reduction in softness of patellar ligament in dogs affected by CCL injuries [21]. A recent paper described increased hardness of the entire ligament in dogs undergoing TPLO and TTA. However, the authors did not investigate the structural and elastic changes in the patellar ligament at different times after tibial osteotomy [29].

The first aim of this study is to assess the morpho-functional changes in the patellar ligament through radiographic, ultrasonographic and elastosonographic evaluations of dogs undergoing TPLO for cranial cruciate ligament rupture during the first six months of the postoperative follow-up. The second purpose is to correlate the radiographic and conventional ultrasonography changes in the patellar ligament with its elastic properties assessed through the real-time strain elastosonography.

## 2. Materials and Methods

### 2.1. Data Collection

In this prospective clinical study, we enrolled dogs weighing between 20 and 50 kg, regardless of breed or sex, undergoing TPLO following CCL rupture at the University Teaching Veterinary Hospital.

Only healthy, non-pregnant patients with a unilateral CCL rupture and without another orthopedic pathology affecting the pelvic limbs were enrolled in this study.

The diagnosis of complete CCL rupture was based on a positive cranial drawer test and/or tibial compression test.

The Ethics Committee for Clinical Studies on Animal Patients of the University of Camerino, Italy, with the protocol number 1/2024, approved this study, and all owners gave specific informed consent.

### 2.2. Surgical Procedure

The dogs were positioned in dorsolateral recumbency, and craniomedial approach to the proximal tibia was obtained through sartorius muscle elevation.

After the partial detachment of the popliteus muscle from the caudal portion of the tibia and the application of a Jig, radial osteotomy was performed with a dedicated saw, and the proximal portion of the tibia was rotated to achieve an angle of tibial plateau between 2° and 5°. A 1.2 mm Kirschner wire was placed proximal to Sharpey’s fibers at the attachment of the patellar ligament (about 0.5 cm of patellar ligament attachment) across the osteotomy and through the caudal cortex of the proximal tibia to temporarily stabilize the limb.

DePuy Synthes TPLO Locking Plates (DePuy Synthes, Oberdorf, Switzerland) were applied according to the size of the patient.

No arthrotomy or arthroscopy was performed. During the surgery, the researchers administered intravenous cefazoline 30 min before the skin incision and performed a second administration after 90 min at a dosage of 22 mg/kg. The dogs received cefalexin (20 mg/kg) every 12 h for five days postoperatively and carprofen (3 mg/kg) every 24 h for eight days after the surgery. Physical activity restriction was prescribed until radiographic bone healing.

### 2.3. Radiographic Evaluation

The researchers performed radiographic examinations of the stifle from mediolateral and caudocranial views before the surgery (T0) and at one month (T1), two months (T2) and six months (T3) after the surgery.

Radiographic examination was performed under sedation using digital direct equipment (FDR D-EVO II, Fujifilm, Milano, Italy).

The mediolateral view was obtained with the stifle and hock joints flexed at 90°. The researchers evaluated the thickness of the patellar ligament from the mediolateral view at three points: proximal, central and distal.

**Figure 1:**
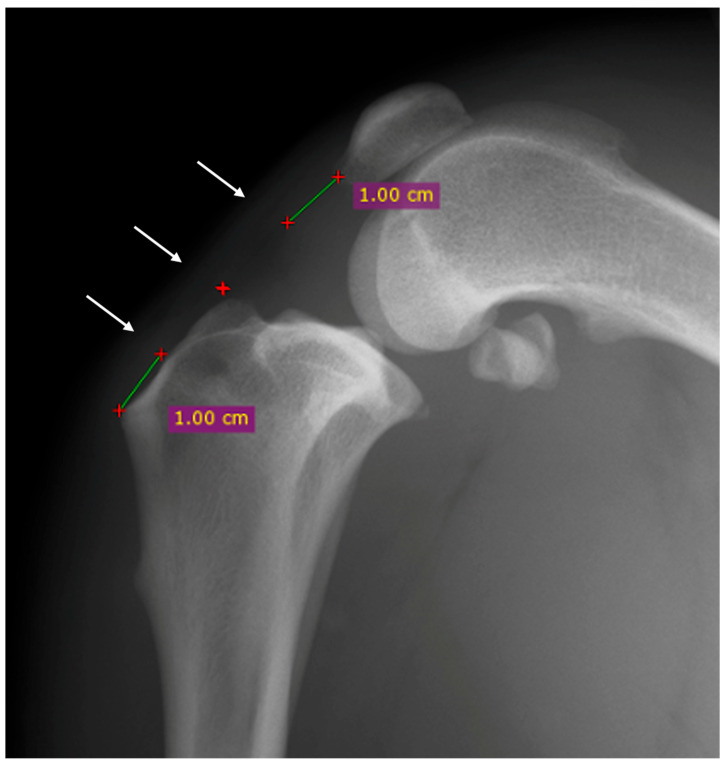
Mediolateral radiographic view of the stifle before the surgery. The thickness of the patellar was evaluated at three points: proximal, central and distal (white arrows). The proximal point was established one centimeter distally to the base of the patella (green line), the distal point was established one centimeter proximally to the tibial tuberosity (green line) and the central point was halfway between the first two points previously described (red cross).

The proximal point was established one centimeter distally to the base of the patella, the distal point was established one centimeter proximally to the tibial tuberosity, and the central point was halfway between the first two points previously described (Figure 1) [16].

Evaluations were performed at T0, T1, T2 and T3.

### 2.4. Ultrasonographic and Strain Elastosonographic Evaluations

The researchers performed the ultrasonographic and elastosonographic examinations at T0, T1, T2 and T3.

They positioned the dogs in lateral recumbency, with the affected limb uppermost and the stifle in maximal passive flexion as previously described [28]. A MyLab Class C ultrasound machine (Esaote, Genova, Italy) equipped with a 12–18 MHz linear transducer (LA 435; Esaote, Genova, Italy) was used for the ultrasonographic assessment.

The ultrasound thickness of the patellar ligament was evaluated using longitudinal scansion.

**Figure 2:**
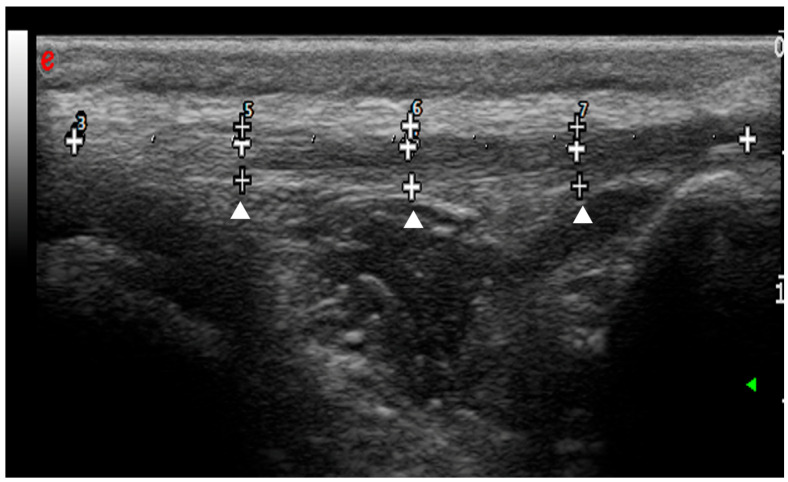
Ultrasound longitudinal scansion of the patellar ligament before the surgery. The total length of the patellar ligament was divided into four equal segments (white crosses). The measurements of patellar ligament thickness were conducted at the junction between the segments to obtain three different points: proximal, central and distal (arrowheads).

The total length of the patellar ligament (from the patella to the tibial tuberosity) was divided into four equal segments, and measurements were conducted at the junction between the segments to obtain three different points: proximal, central and distal (Figure 2).

The radiographic and ultrasonographic patellar ligament values were normalized as a percentage ratio (%) of patellar ligament thickness (mm) to the tibia width (mm). The tibia width was measured from the distal end of the tibial crest to the most caudal point of the caudal cortex perpendicular to the cranial border from a mediolateral radiographic view.

Moreover, the ultrasonographic assessment of the patellar ligament was also based on echogenicity and the fiber architecture and was performed using the following scoring system:

0 = normal thickness, shape, echogenicity and fiber architecture; 1 = ligament thickening with normal echogenicity and fiber architecture; 2 = ligament thickening with fiber disruption, changes in echogenicity and periligamentous hyperechogenicity; and 3 = ligament thickening with distinct anechoic core lesions (i.e., complete absence of fiber echos) surrounding fiber disruption and periligamentous hyperechogenicity or edema.

The elastosonographic exam was performed by the same operator with the same probe and machine applying rhythmic and compressive movements with the probe. The color map of the superimposed B-mode images indicates the different elasticities of tissue. Specifially, the hard tissue is blue, the intermediate is green and the soft tissue is red.

The percentages of hardness (HRD) and softness (SFT) were calculated using dedicated software (ElaXto, Esaote). The region of interest (ROI) was considered the entire main body of the ligament, excluding its origin in the distal patella and tibial tuberosity. Only the longitudinal scansion was examined. The quality indicator of the elastosonographic images was the presence of a green coilin spring and the blue coloration of the skin and dermal tissue.

### 2.5. Statistical Analysis

A simple size calculation was performed considering a previous study that evaluated the thickness of the patellar ligament in dogs [16]. Power calculation was conducted using G*Power software (version 3.1.9.6; University of Düsseldorf, Germany) for repeated measures ANOVA with a power of 0.90, an alpha error of 0.05, and an effect size f = 0.7861984. This suggested that a minimum of 18 dogs would have been sufficient to detect significant differences.

The data were summarized using the arithmetic mean and standard deviation (SD) and were assessed for the assumption of normality of the data distribution using the Shapiro–Wilk test. The normally distributed cardinal data were analyzed with repeated measures ANOVA (Analysis of Variance) and the Holm–Šidák post hoc test; otherwise, statistical analyses were performed with the Friedman test and Dunn’s multiple comparison test. Differences with *p* values < 0.05 were considered statistically significant.

The relationship between the mean normalized thickness of the patellar ligament, measured radiographically and ultrasonographically, and the elastosonographic properties of the patellar ligament, measured as percentages of hardness and softness, was analyzed by Spearman’s correlation and quantified with the coefficient *r*s in the correlation matrix. The coefficient *r*s was considered significant if it was higher than the critical value of *r*s for *p*-values below 0.05.

All the data were analyzed using GraphPad Prism 10 statistical software for MacOS, version 10.1.1-270 (GraphPad Software Inc., San Diego, CA, USA).

## 3. Results

### 3.1. Enrolled Patients

Thirty patients were assessed for eligibility, but three were excluded because of a bilateral cranial cruciate rupture. Five subjects were then excluded for having an incomplete follow-up.

**Figure 3:**
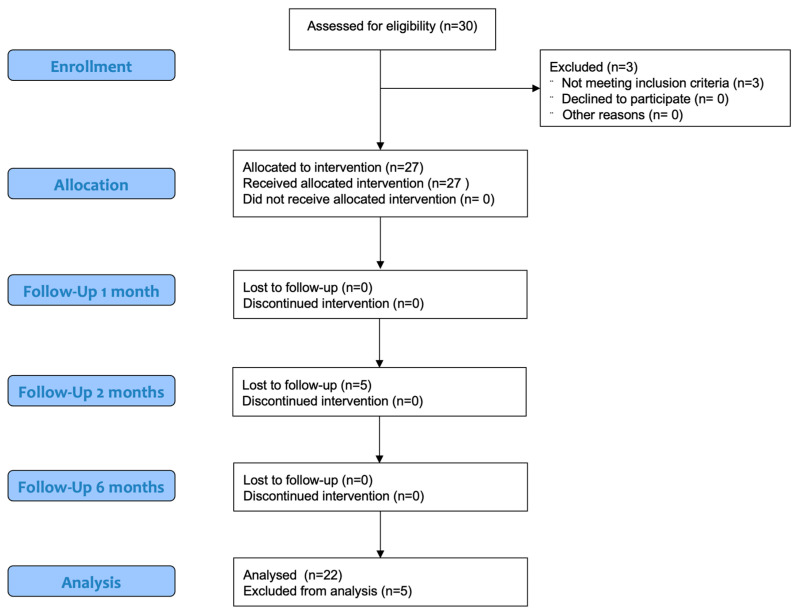
Modified CONSORT flow diagram for subjects included in clinical trial.

Twenty-two dogs completed the trial. This paper conforms to the Consolidated Standards of Reporting Trials (CONSORT) Statement 2010 for reporting randomized clinical trials (Figure 3) [30]. Different breeds were enrolled: three Golden Retrievers, two Labrador Retrievers, two American Staffordshire Terriers, two Siberian Huskies, two American Pit Bull Terriers, two Rottweilers, one Cane Corso, one Deutscher Boxer, one Braque Saint-Germain, one German Shepherd, one Bracco Italiano, one Australian Shepherd and three mixed-breed dogs. The main ± SD weight and age of patients at T0 were 29.9 ± 9.01 kg and 4.6 ± 2.7 years, respectively.

The main ± SD preoperative tibial plateau angle (TPA) was 26.6 ± 2.21°, and the postoperative TPA was 5 ± 2.2° (15 stifles showed a TPA less or equal to 5°, 4 dogs had a TPA of 6°, 2 dogs 8° and 1 had 10°).

All the patients had a regular follow-up and had no complications.

### 3.2. Radiographic and Ultrasonographic Evaluations

At T0, the patellar ligament in the proximal, central and distal portions showed statistically reduced radiographic thicknesses compared to those at T1, T2 and T3. No statistical differences were highlighted between T1, T2 and T3 (Table 1). The comparison between these three points at the same time in this study (proximal, central and distal) showed no statistical differences at the baseline; however, it showed statistical differences at T1 between all the three points (proximal–distal, proximal–central and central–distal) and at T2 and T3 just between proximal–distal and proximal–central.

**Figure 4:**
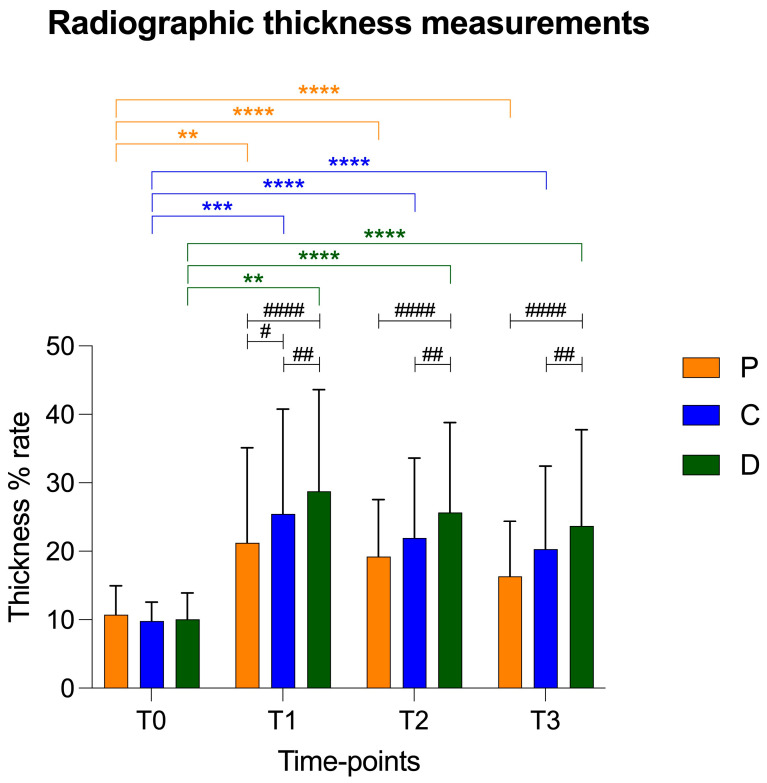
Graphical representation of radiographic patellar ligament thickening at T0, T1, T2 and T3 at proximal (P), central (C) and distal (D) measurement points. Asterisks colored according to measurement point indicate significant differences between time-points within each measurement point. Hashtags indicate significant differences between measurement points at each time-point. *p*-values, #: *p* < 0.05; **/##: *p* < 0.01; ***: *p* < 0.001; ****/####: *p* < 0.0001.

There was a statistical increase in thickness in the central and distal points compared to that of the proximal point at T1. In addition, the patellar ligament in the distal portion was statistically thicker in comparison to the proximal and central points at T2 and T3 (Figure 4). The trend of the ultrasonographic results was similar to radiographic thickness of the patellar ligament.

**Figure 5:**
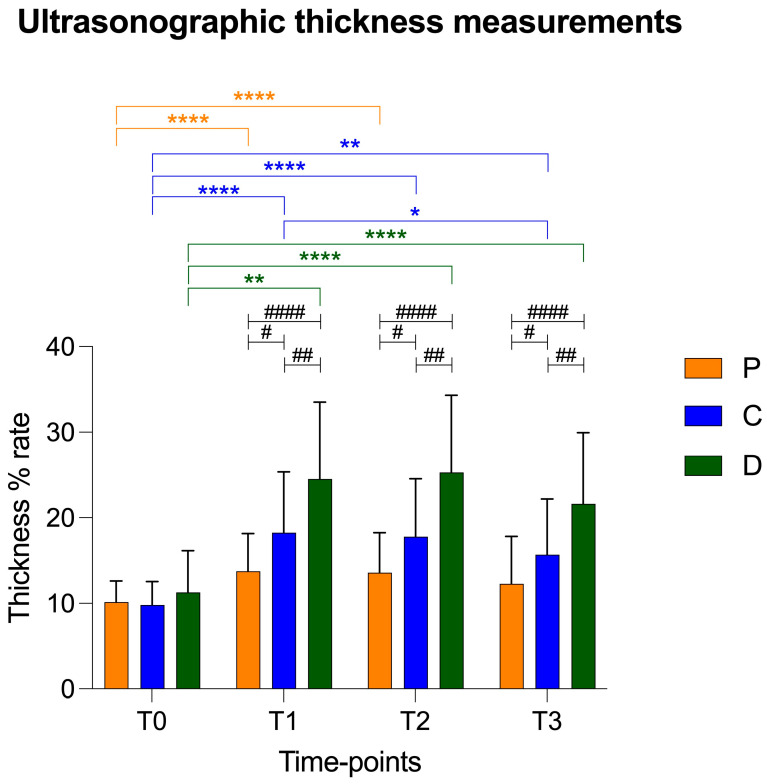
Graphical representation of ultrasonographic patellar ligament thickening at T0, T1, T2 and T3 at proximal (P), central (C) and distal (D) measurement points. Asterisks colored according to measurement point indicate significant differences between time-points within each measurement point. Hashtags indicate significant differences between measurement points at each time-point. *p*-values, */#: *p* < 0.05; **/##: *p* < 0.01; ****/####: *p* < 0.0001.

The values of the patellar ligament thickness are reported as mean ± standard deviation in Table 2 and in Figure 5.

At T0, all the subjects presented with a patellar ligament with slight morphological and structural modifications classified by ultrasound as grade 1. At T1 and T2, 82% of the patellar ligaments had moderate abnormalities, including fiber disorganization, changes in echogenicity and periligamentous hyperechogenicity (score = 2), while the remaining 18% showed slight modifications (score = 1). At the last time-point (T3), the echogenicity and fiber architecture appearance of the ligament were slightly similar to the preoperative condition (score = 1); however, they were not exactly the same because five patellar ligaments scored = 3. No subjects showed severe alteration in the patellar ligament aspect (score = 3) (Table 3).

### 3.3. Strain Elastosonographic Evaluation

At T1 (HRD = 52.29 ± 26.98%; SFT = 45.95 ± 26.12%), T2 (HRD = 58.25 ± 23.87%; SFT = 44.05 ± 23.64%) and T3 (HRD = 47.95 ± 23.59%; SFT = 52.13 ± 24.40%), there were increased hardness and a reduction in softness of the patellar ligament compared to those at T0 (HRD = 25.86 ± 25.61; SFT = 74.06 ± 25.68). At T3, the hardness values showed a tendency to decrease, but they showed no statistical difference at T2 and T1.

**Figure 6:**
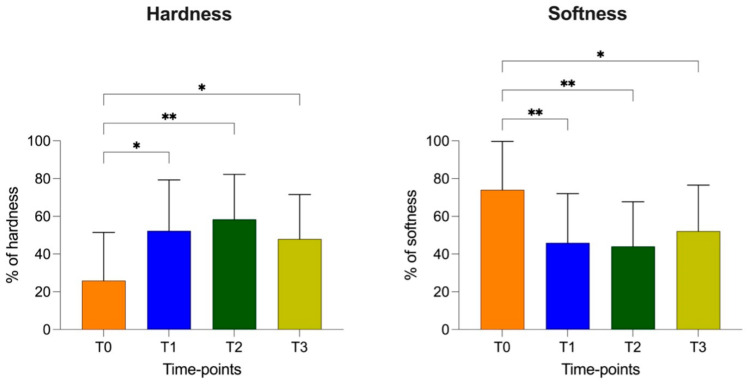
Elastic properties (percentage of hardness and softness with standard deviation) of patellar ligament at T0, T1, T2 and T3. *p*-values, *: *p* < 0.05; **: *p* < 0.01.

**Figure 7:**
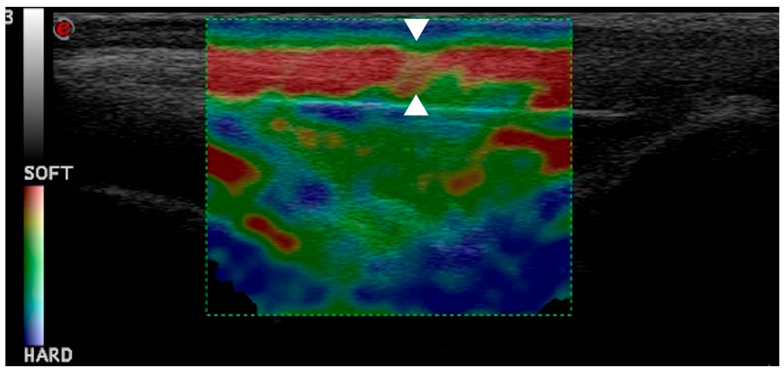
This elastogram shows green and yellow areas with red coloring, indicating hardening of patellar ligament one month post-TPLO (arrowheads).

The softness values showed a similar opposite trend, which increased, but not as significantly compared to the previous times (T2 and T1) and without reaching the value at T0 (Figure 6 and Figure 7).

### 3.4. Correlation Between Radiographic, Ultrasonographic and Elastosonographic Measurements

Spearman’s correlation showed a significant relationship between the RX thickness mean and the US thickness mean (*r*s = 0.74; *p* < 0.0001).

**Figure 8:**
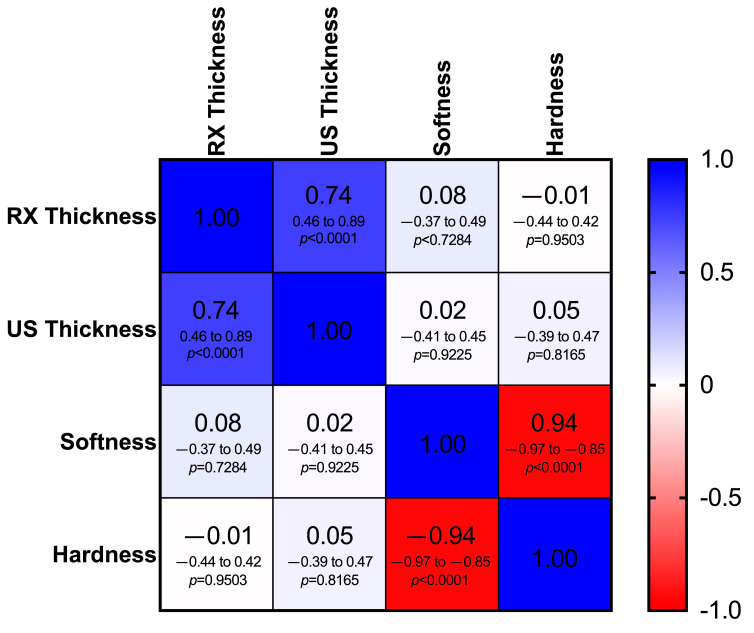
Heat map of the correlation matrix showing the relationship between mean thickness of the patellar ligament measured radiographically (RX thickness) and ultrasonographically (US thickness) and elastosonographic properties (percentage of hardness and softness) of the patellar ligament. Different shades of blue indicate a positive correlation, while different shades of red indicate a negative correlation. In each cell, the Spearman correlation coefficient (*r*s), the 95% confidence intervals (95% CI) and the *p*-value (*p*) are shown on the first, second and third lines, respectively (critical value of *r*s = 0.361).

No correlation was highlighted between the elastosonographic analysis and the RX thickness mean and the US thickness mean (*p* > 0.05). A negative correlation was displayed between HRD% and SFT% (Figure 8).

## 4. Discussion

With regard to patellar ligament tendinopathy following TPLO, the first detectable sign is ligament thickening [31]. The additional imaging findings include soft tissue swelling, periligamentous hyperechogenicity and alteration in the normal fiber architecture [16,32]. In accordance with the previous literature, our radiographic and ultrasonographic results showed an increase in thickness of the patellar ligament at one, two and six months post-TPLO [15,16,17]. At six months, the thickness of the ligament was slightly reduced compared to the ligament at the previous follow-ups; however, the values were significantly higher than those in the preoperative period. Although at the baseline there were no significant differences in patellar ligament thickness at the three points (proximal, central and distal), the subsequent three postoperative follow-ups revealed the most thickening in the most distal portion of the ligament. This statistical difference observed in both the radiographic and ultrasonographic measurements may be attributed to the trauma induced by the saw during osteotomy or the thermal damage of anti-rotational pin application, as previously reported in the literature [11,12,18,33,34,35]. It should be noted, however, that the surgeon was always very careful not to touch the ligament with the saw and to cool the pin with saline during insertion during the osteotomy. Probably, according to a translational study, an alteration in articular biomechanics is the most reliable theory [18]. The point of joint rotation on the femur is cranially translated relative to the tibia following TPLO. In this way, the lever arm of the femorotibial joint is reduced, requiring a greater vector force to achieve stifle extension. The force is provided by the quadriceps muscle during the extension of the stifle, and an increase in this force may have overstressed the distal insertion of the patellar ligament at the tibial tuberosity, causing degenerative changes [18].

An ex vivo study reported a significant increase in patellar ligament strain with a TPA between 0° and 5° after TPLO [35]. On the contrary, the patellar ligament strain measured post-TPLO at a TPA of 10° was similar to that of the pre-TPLO intact CCL stifle. However, the authors did not consider the deformation of the quadriceps muscle that reduces the strain on the patellar ligament [35]. In our research, the mean postoperative TPA was 5 ± 2.2°. Among these, fifteen stifles showed a TPA less than or equal to 5° and seven greater than 5°. Nevertheless, patellar ligament thickening showed the same trend in both the subjects with a postoperative TPA ± of 5° and > of 5°.

We believe that further studies are needed to evaluate the real implication of post-TPLO TPA on the onset of the patellar ligament desmopathy, considering that some studies suggest bringing the post-TPLO TPA to a value lower than 5° to reduce the loading forces on the menisci [36] and improve gait symmetry [37].

Another aspect observed in our study is that during the early postoperative phase, the patellar ligament appeared markedly thickened. Over time, this thickness tended to decrease, without returning to the preoperative conditions. This pattern likely reflects significant alterations induced by rapid biomechanical changes following TPLO, which gradually diminish as the ligament undergoes progressive adaptation.

In this study, a good level of agreement was shown between the radiographic and ultrasonographic values, which means the radiographic thickness of the patellar ligament was comparable with the ultrasonographic measurements. The ultrasonographic investigation, compared to the radiographs, also allowed for the assessment of the fibers’ architecture, the presence of a core lesion and changes in echogenicity. This finding is in agreement with a few studies on the present subject in the literature, which reveal that US images provide superior visualization of the fibrillary patellar ligament architecture and further details regarding the position, extension and paratenon involvement of the fibrillary gap (i.e., tendon fiber discontinuity) [32,38]. According to DeSandre-Robinson et al. (2017), the profile of the patellar ligament is difficult to evaluate in the presence of marked periligamentous soft tissue swelling during a radiographic examination [15]. In our study, the exploration of the stifle joint was not performed with a reduction in tissue reaction, allowing us to easily distinguish the radiographically patellar ligament margins.

The main alterations in the patellar ligament echostructure (echogenicity and fiber architecture) were recorded after one and two months post-TPLO. At six months, there was an improvement in the ligament aspect that appeared similar to the preoperative state; however, 23% of the subjects still presented moderate modification (score = 2). No subject showed severe signs of desmitis, such as distinct anechoic core lesions. These findings are consistent with the trend of patellar ligament thickening.

Traditional ultrasound has been widely used in patellar ligament evaluation in human medicine. Although conventional ultrasound is an essential tool used to evaluate tendon diseases, it only provides information about structural and morphologic changes [39,40]. The assessment of the mechanical properties of tendons is a key point in the diagnosis and treatment of tendinopathy [41].

A complementary ultrasound technique, elastosonography, allows for the evaluation of the tendon’s elasticity related to its functionality [42,43]. Strain and shear wave elastosonography are both used to explore the elasticity of the patellar ligament in human medicine [27]. Palumbo Piccionello et al. (2018) described the elastic properties of the patellar ligament in healthy dogs through strain elastosonography [28]. The results showed that the patellar ligament in clinically normal dogs is highly elastic. Moreover, Pennasilico et al. (2024) reported an increase in rigidity in the patellar ligament in dogs affected by a CCL rupture [21]. The findings of our research are in line with those found in these two previous studies, since at T0 (before the surgery), the patellar ligament of the 22 dogs showed still some softness (SFT of 74.06% ± 25.68), and therefore some elasticity, but with reduced values comparatively with those of the healthy patellar ligaments (SFT of 94.9% ± 9.3%) [28]. This data further confirms that the stifle instability resulting from a CCL rupture in dogs also affects the patellar ligament.

This study and the previous one conducted by the same authors demonstrate that according to the radiographic, ultrasound and elastosonographic investigations, at T0 (before the surgery), the patellar ligament showed the progressive thickening of the ligament in association with moderate abnormalities, including disorganization of the fibers, alterations in echogenicity and periligamentous hyperechogenicity, as well as a reduction in elasticity [21].

Two studies evaluated the stiffness of the patellar ligament in dogs in four different stifle positions. They concluded that the standing position provided a highly soft elastosonogram, and it was the most suitable and reliable stifle position with the least variability [44,45]. In our study, the patellar ligament investigation was performed with the stifle in maximal passive flexion for two reasons; the first was that the patients could have slight lameness in the limb undergoing TPLO in first period postoperatively, and the second was related to the background of the authors [21,28]. Anyway, the elastosonographic evaluation should be performed with the stifle in a standardized position to obtain the most accurate results.

Therefore, the results of these studies may encourage our colleagues to treat patients with a cranial cruciate ligament (CCL) rupture with TPLO as early as possible, to clinically monitor the patellar ligament by diagnostic imaging, and if necessary to implement physical therapy for optimal functional recovery, especially for workers and athletes.

No correlation was highlighted between the radiographic/ultrasonographic and elastosonographic measurements. This is probably because they are based on different assessment methods (patellar thickness versus elastic properties of the ligament). Hardness and softness had a negative correlation; however, this result was predictable because these values are inversely proportional.

This study has some limitations. For example, the results were not supported by the histological and mechanical investigations; however, patients enrolled in clinical studies cannot be subjected to tendon biopsy only for research purposes. Another limitation is the lack of a strain ratio that would have allowed us to perform a semiquantitative evaluation of the patellar ligament compared to the elasticity of surrounding tissue [29]. Del Signore and colleagues (2025) showed that the cutis/subcutis represents a reliable reference tissue for assessing the elastic properties of the patellar ligament in dogs undergoing TPLO or TTA [29]. In addition, as opposed to the radiographic and ultrasound measurements, the elastosonographic evaluations were performed only on the whole ligament, which did not allow us to investigate the different elasticities of the three portions of the patellar ligament.

Based on previous studies, the radiographic thickness of the patellar ligament was evaluated at one centimeter distally to proximal insertion and one centimeter proximally to distal insertion, which could have been influenced by the size of the animals, although the weight variability of the patients was limited, as shown by the standard deviation of weight (29.9 ± 9.01 kg) [16].

## 5. Conclusions

The results of this clinical study highlight the presence of structural and mechanical alterations in the patellar ligament in subjects undergoing TPLO before and over six months after surgery.

The radiographic and ultrasound examinations provide similar results in the evaluation of the thickness of the patellar ligament, while elastosonography did not show a correlation with the other two techniques.

Although this study did not highlight the clinical compliances in the postoperative period, we believe that in the future, it could be useful to clinically monitor patients treated with TPLO, especially through gait analysis and/or plexometric platforms to recognize even minimal clinical anomalies that may be determined by the desmopathy of the patellar ligament and to correlate them to imaging features.

Additionally, it could be interesting compared our data with dogs undergoing early physical rehabilitation following TPLO.

## Figures and Tables

**Table 1:** Mean ± standard deviation in the patellar ligament thickness (percentage of patellar ligament thickness to tibial thickness) measured using X-ray at three points (proximal, central and distal points) at different times (T0, T1, T2 and T3).

Timepoints	Proximal	Central	Distal	Statistics
T0	10.74 ± 4.238 ^a^	9.81 ± 2.76 ^c^	10.06 ± 3.82 ^e^	χ^2^_r_ = 5.545, *p* = 0.0625
T1	21.24 ± 13.91 ^bα^	25.49 ± 15.26 ^dβ^	28.75 ± 14.86 ^fγ^	χ^2^_r_ = 32.48, *p* < 0.0001
T2	19.22 ± 8.36 ^bδ^	21.92 ± 11.67 ^dδ^	25.70 ± 13.11 ^fε^	χ^2^_r_ = 29.73, *p* < 0.0001
T3	16.35 ± 8.05 ^bλ^	20.30 ± 12.14 ^dλ^	23.67 ± 14.10 ^fπ^	χ^2^_r_ = 29.73, *p* < 0.0001
Statistics	χ^2^_r_ = 36.05, *p* < 0.0001	χ^2^_r_ = 38.89, *p* < 0.0001	χ^2^_r_ = 45.65, *p* < 0.0001	

Different pairs of standard superscript letters in the same column indicate significant differences between the time-points and within each point of measurement found using the multiple comparisons test. Different pairs of Greek superscript letters in the same row at each time-point indicate significant differences between points of measurement found using the multiple comparisons test.

**Table 2:** Mean ± standard deviation in patellar ligament thickness (percentage of patellar ligament thickness to tibial thickness) measured using ultrasonography at three points (proximal, central and distal points) at different times (T0, T1, T2 and T3).

Time-Points	Proximal	Central	Distal	Statistics
T0	10.16 ± 2.47 ^a^	9.81 ± 2.76 ^c^	11.28 ± 4.89 ^e^	χ^2^_r_ = 6.475, *p* = 0.1393
T1	13.73 ± 4.43 ^bα^	18.26 ± 7.10 ^dβ^	24.52 ± 9.0 ^fγ^	χ^2^_r_ = 36.64, *p* < 0.0001
T2	13.57 ± 4.67 ^bδ^	17.80 ± 6.77 ^dε^	25.27 ± 9.06 ^fλ^	χ^2^_r_ = 38.27, *p* < 0.0001
T3	12.27 ± 5.54 ^π^	15.67 ± 6.5 ^dτ^	21.63 ± 8.34 ^fγω^	χ^2^_r_ = 36.64, *p* < 0.0001
Statistics	χ^2^_r_ = 31.08, *p* < 0.0001	χ^2^_r_ = 42.93, *p* < 0.0001	χ^2^_r_ = 42.68, *p* < 0.0001	

Different pairs of standard superscript letters in the same column indicate significant differences between the time-points and within each point of measurement found using the multiple comparisons test. Different pairs of Greek superscript letters in the same row at each time-point indicate significant differences between points of measurement found using the multiple comparisons test.

**Table 3:** Ultrasound score of patellar ligaments evaluated at different times (T0, T1, T2 and T3).

Time-Points	Score 0	Score 1	Score 2	Score 3
T0	0/22	22/22 (100%)	0/22	0/22
T1	0/22	4/22 (18%)	18/22 (82%)	0/22
T2	0/22	4/22 (18%)	18/22 (82%)	0/22
T3	0/22	17/22 (77%)	5/22 (23%)	0/22

## Data Availability

The clinical data used to support the findings of this study are included within this article.
